# Characterization of tRNA expression profiles in large offspring syndrome

**DOI:** 10.1186/s12864-022-08496-7

**Published:** 2022-04-07

**Authors:** Anna K. Goldkamp, Yahan Li, Rocio M. Rivera, Darren E. Hagen

**Affiliations:** 1 Department of Animal and Food Sciences, Oklahoma State University, Stillwater, OK USA; 2 Division of Animal Sciences, University of Missouri, Columbia, MO USA

**Keywords:** tRNA, Bovine, Protein translation, Large Offspring Syndrome

## Abstract

**Background:**

Assisted Reproductive Technologies (ART) use can increase the risk of congenital overgrowth syndromes, such as large offspring syndrome (LOS) in ruminants. Epigenetic variations are known to influence gene expression and differentially methylated regions (DMRs) were previously determined to be associated with LOS in cattle. We observed DMRs overlapping tRNA clusters which could affect tRNA abundance and be associated with tissue specificity or overgrowth. Variations in tRNA expression have been identified in several disease pathways suggesting an important role in the regulation of biological processes. Understanding the role of tRNA expression in cattle offers an opportunity to reveal mechanisms of regulation at the translational level. We analyzed tRNA expression in the skeletal muscle and liver tissues of day 105 artificial insemination-conceived, ART-conceived with a normal body weight, and ART-conceived bovine fetuses with a body weight above the 97^th^ percentile compared to Control-AI.

**Results:**

Despite the centrality of tRNAs to translation, in silico predictions have revealed dramatic differences in the number of tRNA genes between humans and cattle (597 vs 1,659). Consistent with reports in human, only a fraction of predicted tRNA genes are expressed. We detected the expression of 474 and 487 bovine tRNA genes in the muscle and liver with the remainder being unexpressed. 193 and 198 unique tRNA sequences were expressed in all treatment groups within muscle and liver respectively. In addition, an average of 193 tRNA sequences were expressed within the same treatment group in different tissues. Some tRNA isodecoders were differentially expressed between treatment groups. In the skeletal muscle and liver, we categorized 11 tRNA isoacceptors with undetected expression as well as an isodecoder that was unexpressed in the liver (Ser^GGA^). Our results identified variation in the proportion of tRNA gene copies expressed between tissues and differences in the highest contributing tRNA anticodon within an amino acid family due to treatment and tissue type. Out of all amino acid families, roughly half of the most highly expressed tRNA isoacceptors correlated to their most frequent codon in the bovine genome.

**Conclusion:**

Although the number of bovine tRNA genes is nearly triple of that of the tRNA genes in human, there is a shared occurrence of transcriptionally inactive tRNA genes in both species. We detected differential expression of tRNA genes as well as tissue- and treatment- specific tRNA transcripts with unique sequence variations that could modulate translation during protein homeostasis or cellular stress, and give rise to regulatory products targeting genes related to overgrowth in the skeletal muscle and/or tumor development in the liver of LOS individuals. While the absence of certain isodecoders may be relieved by wobble base pairing, missing tRNA species could increase the likelihood of mistranslation or mRNA degradation.

**Supplementary Information:**

The online version contains supplementary material available at 10.1186/s12864-022-08496-7.

## Introduction

Assisted Reproductive Technologies (ART) are treatments that increase chances of conception and remedy infertility, which include in vitro fertilization, embryo culture, oocyte in vitro maturation, and embryo transfer [1]. ART is extensively used in human medicine as well as the livestock industry [2–4]. However, the utilization of ART can increase the risk of congenital overgrowth syndromes, such as Beckwith-Wiedemann syndrome (BWS) in humans and large offspring syndrome (LOS) in ruminants [5, 6]. Shared phenotypes between BWS and LOS includes a birth weight above the 97^th^ percentile compared to the general population, an enlarged tongue, umbilical hernia, asymmetrical development, and in humans, an increased chance of tumor development [7–9].

DNA methylation is an epigenetic modification that controls gene expression. We previously identified a dysregulation of (m)RNA transcripts in LOS and regions with aberrant DNA methylation, and some of these regions were associated with loss of imprinting at imprinted domains [10]. Distinct sets of tissue-specific methylation patterns in cattle have been described, yet the regulatory impacts of these regions are poorly understood [11]. A report found that 24.59% and 22.43% of transfer RNA (tRNA) genes are methylated in fetal and adult bovine muscle tissue [12] and elevated methylation levels at tRNA gene clusters have been identified which resulted in transcriptional repression and demonstrated that epigenetic mechanisms can fine tune tRNA expression [13]. In addition, recent tRNA studies in human cancer have indicated that DNA methylation interferes with the binding of the transcriptional machinery (RNA polymerase III & TFIIIC) to the promoter of tRNA genes and inhibits tRNA expression [14]. We have observed DNA methylation overlapping tRNA gene clusters [10]. This suggests differential methylation of tRNA genes may lead to altered spatio-temporal tRNA expression in epigenetic disorders, such as bovine LOS. However, the mechanisms underlying tRNA availability and its relationship to tissue specificity or overgrowth has not been investigated.

Due to their crucial role in translation, tRNAs were once thought to be ubiquitously expressed in all tissues and species. tRNA genes can be classified as isoacceptors or isodecoders. tRNA isoacceptors have different anticodons but are charged with the same amino acid whereas tRNA isodecoders have the same anticodon and amino acid but with sequence differences within the body of the tRNA (outside of the anticodon) [15]. Nearly half of human tRNA genes are in a transcriptionally silent state with many unexpressed genes encoding isodecoders with the same translational capacity [16]. An elevation of tRNA levels has been identified in several cancer types, acting as a mechanism to promote tumor growth and angiogenesis [17–20]. For example, nuclear- and mitochondrial- tRNAs have pronounced expression profiles in breast cancer, revealing that they can be used as biomarkers [21]. Overexpression of the initiator methionine tRNA (tRNA_i_^Met^) in cancer has demonstrated the increased abundance of tRNA_i_^Met^ can influence cell metabolic activity and increase metastatic potential [22, 23]. Codon optimality and the availability of tRNAs in the cytoplasmic pool can mediate the degradation of mRNA transcripts due to low translational efficiency [24]. In addition, tRNAs have been observed to act as a source of small non-coding RNA, called tRNA-derived fragments, which participate in gene regulation through full or partial complementarity to mRNA transcripts [16, 25–30]. Considering that changes in the epigenome have been found to influence gene expression, differentially methylated tRNA genes could affect tRNA abundance and protein expression which could be linked to tissue specificity or an overgrowth phenotype. The complexities of tRNA expression and their alternative functions have been overlooked and there is a need to extend tRNA studies across species.

In this study, we utilized high-throughput tRNA sequencing to investigate tRNA expression in skeletal muscle and liver tissue of day 105 artificial insemination-conceived (Control-AI), ART-conceived with a normal body weight (ART-Normal), and ART-conceived bovine fetuses with a body weight above the 97^th^ percentile compared to Control-AI (ART-LOS). This study represents the first in-depth assessment of tRNA expression across tissues in cattle and in congenital overgrowth syndrome.

## Results

### Conservation of tRNA genes across species

**Fig. 1:**
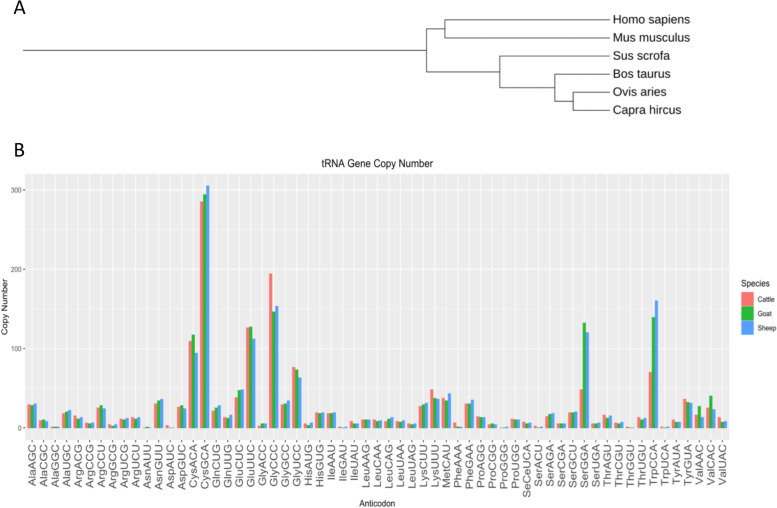
Summary of tRNA gene conservation across different species. (**A**) Phylogenetic tree of the evolutionary relationship between *Mus musculus* (mouse; GRCm38.p4), *Homo sapiens* (human; GRch38.p12), *Sus scrofa* (pig; Sscrofa11.1), *Bos taurus* (cow; ARS-UCD1.2), *Ovis aries* (sheep; Oar_rambouillet_v1.0), and *Capra hircus* (goat; ARS1). Tree was produced using the phyloT web server, based on NCBI taxonomy. Scientific name is listed for each species. (**B**) Distribution of annotated gene copy numbers grouped at the level of the anticodon in the reference genomes of *Bos taurus* (cattle), *Ovis aries* (sheep), and *Capra hircus* (Goat)

Through computational prediction using tRNAscan-SE (http://gtrnadb.ucsc.edu), 1,659 annotated tRNA genes have previously been identified within the cattle reference assembly, ARS-UCD1.2 [31]. Of these annotated tRNA genes, 1,637 are encoded in the nuclear genome and 22 tRNAs are mitochondrially-encoded. We find that the number of tRNA gene copies between species is extremely variable. Compared to the human reference genome (GRch38.p12), there are three times as many computationally predicted tRNA genes in the bovine genome (597 vs 1,659). Despite large disparities in the number of tRNA genes between humans and cattle, conservation of tRNA numbers in the context of evolution across closely related species is observed: murine (422 tRNAs; GRCm38.p4), swine (510 tRNAs; Sscrofa11.1), ovine (1,774 tRNAs; Oar_rambouillet_v1.0), and caprine (1,770 tRNAs; ARS1) (Fig. **1**A). The conservation of tRNAs suggests there were a series of duplications in tRNA gene clusters since the divergence of cow, goat, and sheep that resulted in a tRNA gene expansion within ruminant species (Fig. **1**A).

Codons are considered degenerate because there is a maximum of 61 possible triplet codes, encoding 20 amino acids across species [32]. Therefore, the inflation of tRNA gene copy number in ruminants compared to other species is due to redundancy of tRNA genes. To further investigate the patterns of gene copy number conservation across ruminants, we summarized the number of annotated tRNA genes for each anticodon across three ruminant reference genomes (ARS-UCD1.2, Oar_rambouillet_v1.0, and ARS1) (Fig. **1**B). Two tRNA isodecoders (Leu^GAG^ and Val^CAG^) were unannotated in the bovine, ovine, and caprine assemblies and therefore were not included. In addition, we also found that there were no annotations for Asn^AUU^ and Pro^GGG^ in bovine. The number of gene copies for the tRNAs within each ruminant species are included in Table S2. There are no high confidence predictions for any of the 4 tRNAs in the older (UMD3.1) or current (ARS-UCD1.2) versions of the bovine genome according to tRNAscan-SE. Leu^GAG^, Val^CAG^, Asn^AUU^ and Pro^GGG^ genes are classified as either pseudogenes and/or as “secondary filtered” because they had low feature scores. This scoring system helps to classify if tRNAs are functional in translation and accounts for tRNA-derived short interspersed repeated elements (SINEs) [33]. A recent review summarized “missing tRNA genes” across different kingdoms, illustrating that Pro^GGG^, Leu^GAG^ and Val^CAG^ are absent in 60 eukarya species whereas Asn^AUU^ is absent from 60 eukarya, 100 bacteria, and 50 archaea species [34].

Variation in tRNA expression across and within treatment groups was assessed using principal component analysis (PCA) and relative log expression (RLE) plots (Supplementary Fig. 1 & 2). Control-AI vs ART-Normal (Supplementary Fig. 1A & 2A) and Control-AI vs ART-LOS (Supplementary Fig. 1B & 2B) PCA plots display high diversity and reduced clustering. However, we find the least amount of variation between ART-Normal and ART-LOS groups in muscle (Supplementary Fig. 1C) and liver tissues (Supplementary Fig. 2C). These results are consistent with a previous study, in which tRNAs did not tightly cluster in Archaea, Bacteria, and Eukarya [35].

### Overview of tRNA sequencing data

We capitalize on the use of an overgrowth syndrome in order to identify tRNA expression profiles in a specific stage of development and condition in skeletal muscle and liver tissue. Tissues collected from Control-AI, ART-Normal, and ART-LOS day 105 bovine fetuses were subjected to YAMAT-seq (n = 13 animals per tissue). YAMAT-seq utilizes specialized Y-shaped adapters to specifically bind to the CCA tail and discriminator base of the tRNA molecule. The YAMAT adapter sequences were removed from the raw sequence reads, which yielded a total of 56,766,658 and 52,642,497 across all samples in muscle and liver. This averaged to 4,366,666 (79.05%) and 4,049,423 (91.2%) reads retained per sample for skeletal muscle and liver, respectively (Table S1A). The trimmed reads were then aligned to the ARS-UCD1.2 bovine reference genome with Hisat2. Across all samples, 395,858,931 and 269,716,390 total reads aligned to nuclear tRNAs in muscle and liver. Contrastingly, 7,455,401 and 13,541,752 total reads aligned to mitochondrial (MT) tRNA genes (Table S1B & S1C). Overall, an average of 30,450,687 (98.15%) and 20,747,415 (95.22%) reads per sample aligned to nuclear tRNAs and an average of 573,492 (1.85%) and 1,041,673 (4.78%) reads per sample aligned to MT tRNAs in muscle and liver. Given that the majority of tRNA genes (1,637 out of 1,659) are nuclearly-encoded, it is expected that a large proportion of reads originate from the nucleus.

### Assessment of unique and shared tRNA sequences

Of the 1,659 tRNA genes annotated in the cattle reference assembly, 1,159 of the muscle and 1,155 of the liver tRNA genes were not expressed in any of the samples (CPM = 0) (Table S3A, S3B). We included a filtering step, in which tRNAs with counts present in any two individuals within a tissue (*n* = 13) were classified as expressed and kept for analysis, yielding a total of 474 and 487 tRNA genes expressed within at least one treatment group in the muscle and liver respectively.

### Expression within each treatment group

In the Control-AI treatment group, 476 tRNA genes were expressed in the liver and 468 tRNA genes were expressed in the muscle. In the ART-LOS group, 468 tRNA genes were expressed in the liver and 466 tRNA genes were expressed in the muscle. In the ART-Normal group, 476 tRNA genes were expressed in the liver and 459 tRNA genes were expressed in the muscle.

### Expression across treatment groups and tissues

**Fig. 2:**
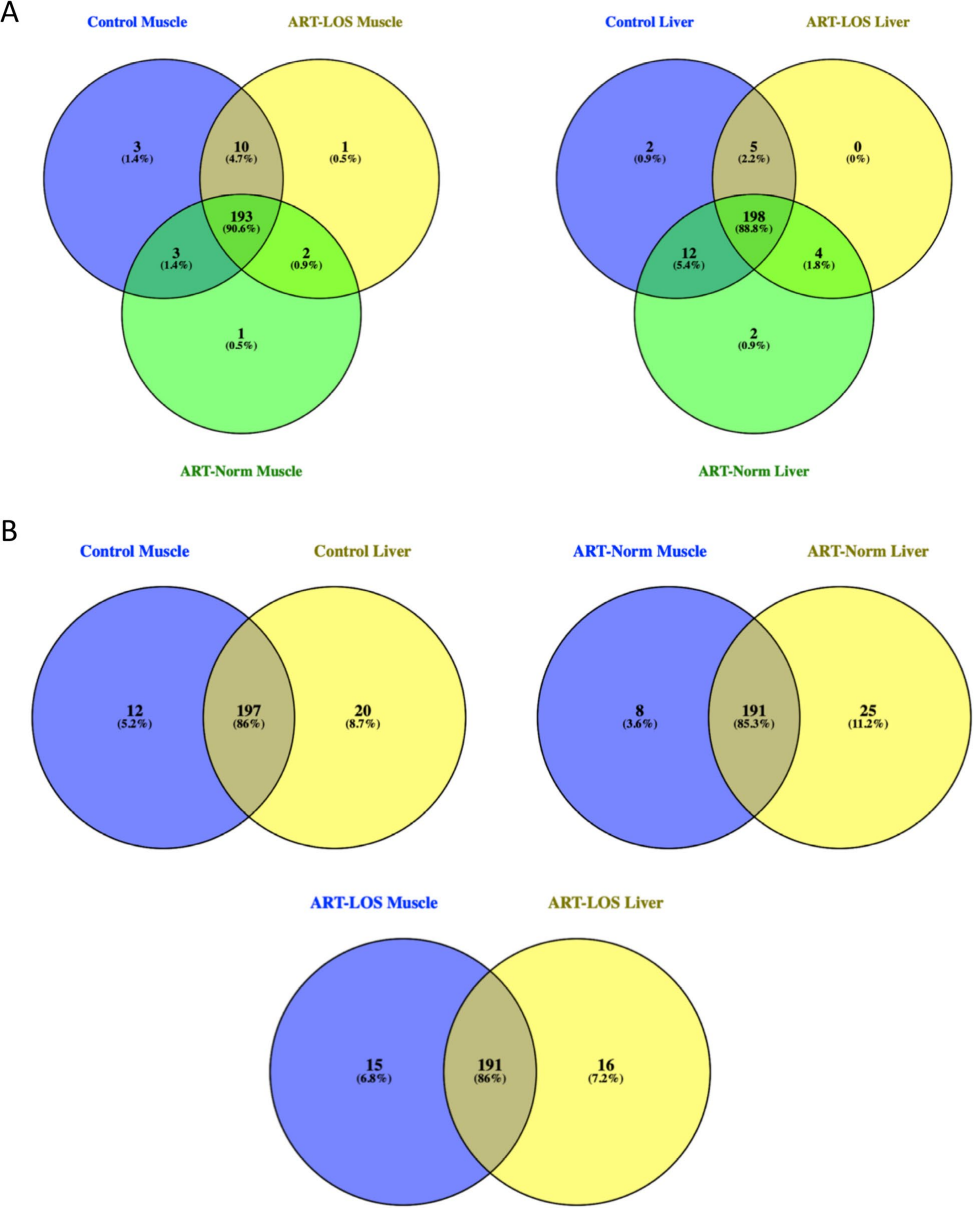
Unique and shared tRNA sequences. tRNAs were classified based on sequence differences and considered to be unique if they were expressed in only one tissue or treatment group(s). The percentages shown indicate the proportion of expressed tRNA sequences that are shared or unique to a particular tissue or treatment. (**A**) tRNA sequences present in different treatments within muscle and liver. (**B**) tRNA sequences shared and uniquely expressed between the same treatment group in a different tissue

Eukaryotic tRNA isodecoders can be transcribed from numerous genomic loci, some of which produce tRNAs with entirely identical sequences [15, 36]. Of the 1,659 tRNA loci, only 1,339 genes encode unique sequences differing by one or more bases. Due to the redundancy of tRNAs in the genome and the inability to decipher the origin of tRNA transcripts bearing identical sequences, we classified tRNAs by sequence instead of by genomic location. Unique tRNA sequences were identified that were expressed in a particular tissue or treatment group (Fig. **2**A-B). These tRNA sequences were classified as “unique” if they were not expressed in the other treatment group(s) or tissue type. We found an average of 85.8% of tRNAs were expressed in the same treatment group between muscle and liver tissue with the remainder being expressed in a tissue specific manner (Fig. **2**B). In addition, 90.6% and 88.8% of tRNA sequences had shared expression across all treatment groups within the muscle and liver respectively (Fig. **2**A). Because some of these unique tRNA sequences could be transcribed from several genes, each possible gene ID was included for the respective sequence and can be found in Table S4.

### Association between tRNA isodecoder abundance and gene copy number

**Fig. 3:**
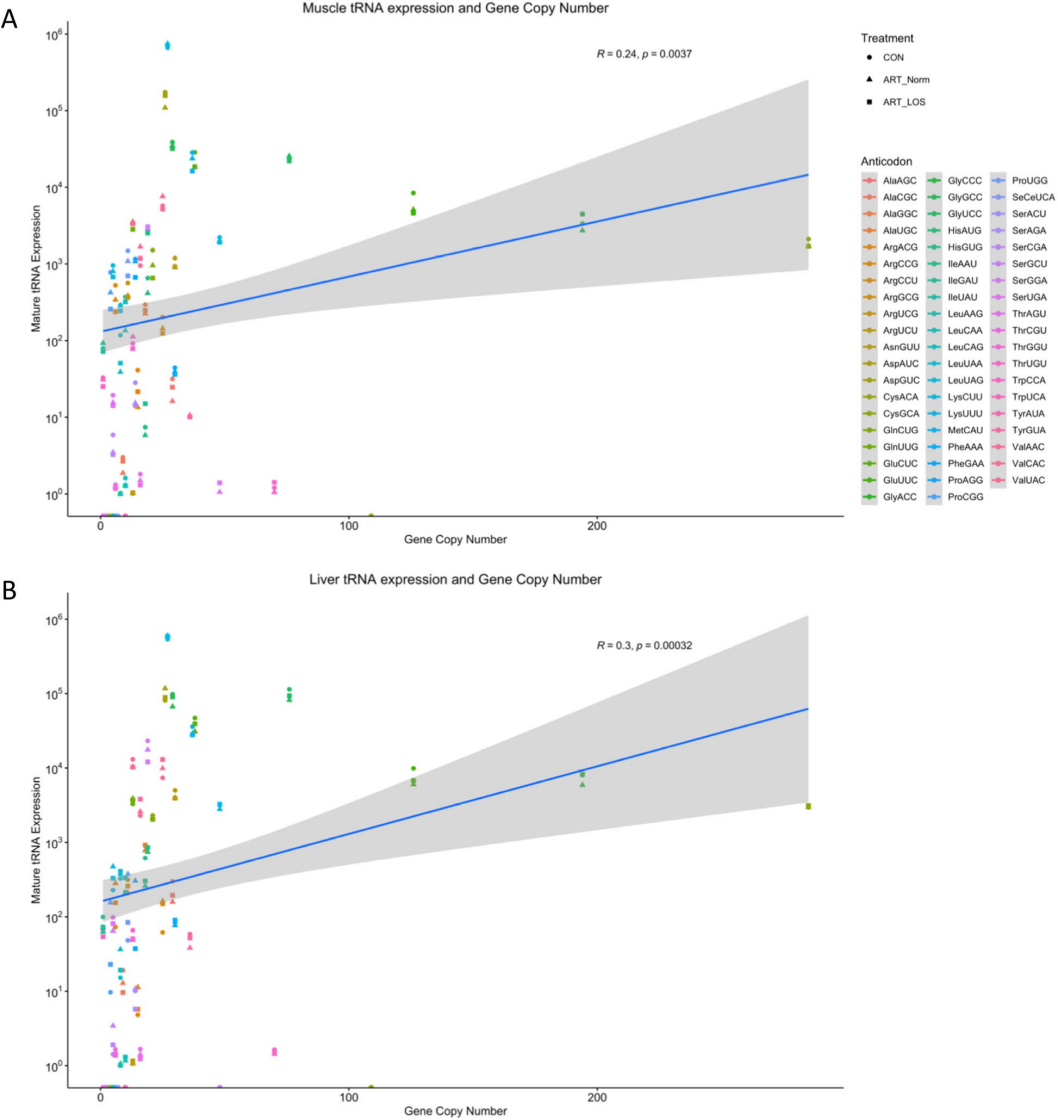
Correlation of mature tRNA expression with gene copy number at the level of the anticodon. A scatterplot showing the relationship of CPM values (y-axis) versus copy number value (x-axis) across all annotated tRNA genes in (**A**) muscle and (**B**) liver. Test based on Pearson’s product moment correlation coefficient and follows a t-distribution with length(x)-2 degrees of freedom if the samples follow independent normal distributions. In order to add a regression line, the geom_smooth() function of ggplot2 was used with the linear model argument method

Although all tRNA genes were once thought to be expressed equally, recent studies have identified variations in tRNA isodecoder expression as well as the presence of tRNAs with undetectable expression [16, 37]. In order to determine if there was an association between tRNA expression and the number of tRNA genes, we performed a Pearson correlation analysis between tRNA expression and tRNA gene copy number. We found that the Pearson correlation coefficients fell below 0.4 for muscle (*R* = 0.24; *p* = 0.0037) and liver (*R* = 0.3; *p* = 0.00032) (Fig. **3**A-B). Although this analysis demonstrates significance, we did not find a strong positive correlation between copy number and expression of tRNAs, suggesting tRNA abundance is independent of gene copy number and is subject to selective transcription in response to treatment and tissue type. Evidence for this is supported by removing 4 tRNAs with high gene copy numbers (Cys^GCA^, Gly^CCC^, Glu^UUC^, Gly^UCC^), in which the correlation became non-significant in the muscle and was unchanged in the liver (Data not shown). These results suggest tRNA gene copy number is not a good proxy for tRNA expression and quantification of tRNA abundance is crucial for determining codon optimality.

**Fig. 4:**
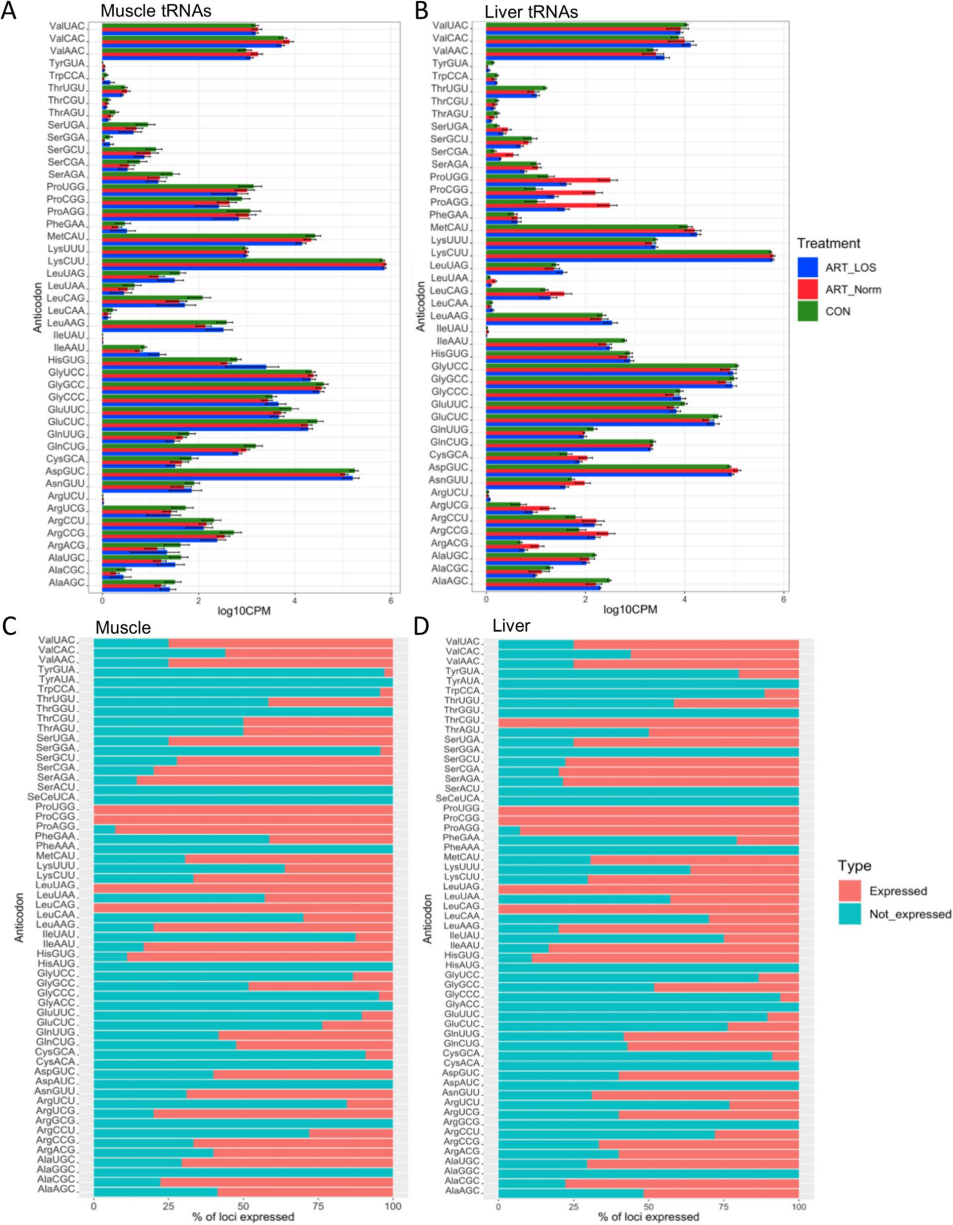
Nuclear and mitochondrial tRNA expression profiles. tRNA abundance across Control-AI, ART-Normal, and ART-LOS in (**A**) muscle and (**B**) liver. Standard error bars are shown for each anticodon and treatment group and were computed with the SummarySE function of Rmisc. Proportion of isodecoder loci expressed within the control individuals for (**C**) muscle and (**D**) liver. Each tRNA species is grouped at the level of the anticodon and the number of expressed copies was divided by total isodecoder copies annotated in the bovine genome to calculate percentage expressed

The observation that tRNAs are selectively expressed, led us to hypothesize that there are alterations in tRNA expression levels between tissues and treatment groups. We further characterized tRNA abundance at the level of the anticodon (Fig. **4**A-B). We observed variations in tRNA expression based on tissue, creating unique tRNA profiles to carry out different biological processes within muscle and liver. Several of these variations in expression contributed to differences in tRNA abundance between muscle and liver (Fig. **4**A-B). For example, proline tRNAs in Control-AI are expressed at high levels in the muscle and reduced levels in the liver. Furthermore, treatment-specific variations in tRNA abundance were detected. A histidine tRNA (His^GUG^) displayed increased expression in the muscle tissue of ART-LOS individuals and a number of proline tRNAs (Pro^UGG^, Pro^CGG^, Pro^AGG^) were upregulated in the liver tissue of ART-Normal individuals (Fig. **4**A-B). Descriptive statistics of the data were computed using the summarySE function of the Rmisc package. The mean, standard deviation, standard error, and 95% confidence interval for each anticodon can be found in Table S5. EdgeR was used to conduct a differential expression analysis and differentially expressed genes were classified based on sequence instead of chromosomal location.

### Muscle DEG analysis

In a pairwise comparison between ART-Normal and ART-LOS, we identified an upregulation of a tRNA encoding His^GUG^ in ART-LOS individuals. Because this analysis includes tRNAs identical in sequence, there were a number of genomic loci that this tRNA could be transcribed from. In an effort to include all possible origins of transcription, all loci expressing the same tRNA sequence for His^GUG^ are included in Table S6. This result is consistent with our previous findings**,** where we detected elevated levels of His^GUG^ in the muscle tissue of ART-LOS individuals (Fig. **4**A).

### Liver DEG analysis

In a pairwise comparison between Control-AI and ART-Normal, we identified downregulation of a tRNA encoding Glu^UUC^ and upregulation of mitochondrial and nuclear tRNAs (MT-Pro^UGG^, MT-Leu^UAG^, Asp^GUC^, and Lys^CUU^) in ART-Normal individuals. The tRNA sequences, genomic locations, and DEG output can be found in Table S6.

In a comparison between ART-Normal and ART-LOS, there were 4 downregulated tRNA species (Pro^AGG^, Arg^UCG^, Pro^UGG^, and Pro^CGG^) in ART-LOS individuals (Table S6). These results suggest that modulation of tRNA abundance is influenced by method of conception (AI vs ART) and altered development. Variation in tRNA anticodon concentration regulates codon pairing for lowly or highly abundant tRNAs, which could control the efficiency of translation due to tissue specificity, ART use, or overgrowth.

### Differentially methylated regions (DMRs) overlap tRNA genes

Because we observed that certain tRNAs were differentially transcribed and expressed among tissues, we evaluated the relationship between tRNA expression and DMRs in ART-LOS individuals. We previously identified DMRs in the muscle tissue samples used in this study [10]. After mapping these DMRs to the current ARS-UCD1.2 genome assembly we found seven tRNA genes were within 5 kb of three unique DMRs. One DMR directly overlapped the gene body of a tRNA, Ile^AAU^ (GeneID: 112444043). A 5 kb window was selected as we reason these six DMRs may overlap regulatory regions controlling tRNA expression, similar to DMRs in human cancer [14]. However, our DEG analysis did not result in any of the tRNAs within DMR regions being identified as differentially expressed. Given the multicopy nature of tRNAs, it is impossible to determine the exact origin of a tRNA read for tRNA genes with identical sequences. For example, Ile^AAU^ had a gain of methylation in the muscle of ART-LOS individuals. In the bovine genome, there are 18 copies of the Ile^AAU^ gene and the methylated Ile^AAU^ gene shares a sequence identical to 13 tRNA gene loci encoding Ile^AAU^. Therefore, the repetitive characteristics of the tRNA genes within the bovine genome creates difficulties in locating specific transcripts to their gene of origin and, as such, may mask differences in gene expression at any single locus. The seven tRNA genes associated with DMRs are highlighted in Table S3A.

### Selective expression of isodecoder gene copy expression within control-AI tissues

We have identified the correlation between tRNA expression and gene copy number is weak, and have detected variation in tRNA expression within specific tissues and conditions. Given these findings, there can be variation in the proportion of tRNA genes expressed between tissues for a particular anticodon, thus generating differences in the tRNA pool within a cell. In the muscle and liver of Control-AI individuals, we analyzed the proportion of expressed versus unexpressed isodecoder gene copies. Bearing the same anticodon, most isodecoders shared similar gene copy contribution in both tissues. Interestingly, we identified 11 tRNA anticodons with none of their isodecoder gene copies expressed in either the muscle or liver tissues (Ala^GGC^, Arg^GCG^, Asp^AUC^, Cys^ACA^, Gly^ACC^, His^AUG^, Phe^AAA^, Ser^ACU^, SeCe^UCA^, Thr^GGU^, and Tyr^AUA^) (Fig. **4**C-D). We also found that loci encoding Ser^GGA^ were not expressed in the liver, but expressed in the muscle. In addition, there were instances of a reduction or inflation of gene copy use between the two tissues. For example, 80% and 60% of Arg^UCG^ isodecoder copies are expressed in the muscle and liver. In this case, we find differences in the number of expressed loci contributed to an increase in the tRNA pool in the muscle and liver (Fig. **4**A-B). Contrastingly, the proportion of expressed isodecoder gene copies for tRNAs encoding proline (Pro^UGG^, Pro^CGG^, Pro^AGG^) is identical between muscle and liver, yet we find large differences in the abundance of these tRNAs between tissues (Fig. **4**A-B). This result further supports that gene copy number does not necessarily dictate tRNA concentration and actively transcribed tRNAs can be expressed at different levels depending on tissue. The full results of Fig. 4, which includes MT-tRNAs, can be found in Supplementary Fig. 3.

### Tissue- and treatment- specific tRNA isoacceptor contribution

Given the differences in anticodon availability between treatments and/or tissues, we investigated codon usage in the bovine genome and transcriptome to understand if tRNA expression could be explained by codon frequency. The RNAseq datasets from a previous LOS study in the same tissue samples were retrieved in order to detect tissue- or treatment-specific changes in codon usage [10]. We performed a codon usage analysis of all transcripts expressed (≥ 1 TPM) in Control-AI, ART-Normal, and ART-LOS groups in skeletal muscle and liver tissue (Table S7). We compared the relative synonymous codon uses (RSCUs) found in our transcriptome analysis to a reference database summarizing bovine codon frequency (https://www.kazusa.or.jp/codon/) [38]. The results of the transcriptome codon usage analysis revealed that all treatment groups and tissue types shared the same bias for a synonymous codon within each amino acid family, which was consistent with the codon usage frequency database. This may suggest that favorable codons are characterized by those that correspond to abundant tRNAs present in the cell. The preferred synonymous codon in each amino acid family is as follows: Ala^GCC^, Arg^CGG^, Asn^AAC^, Asp^GAC^, Cys^TGC^, Gln^CAG^, Glu^GAG^, Gly^GGC^, His^CAC^, Ile^ATC^, Leu^CTG^, Lys^AAG^, Met^ATG^, Phe^TTC^, Pro^CCC^, Ser^AGC^, Thr^ACC^, Trp^TGG^, Tyr^TAC^, and Val^GTG^ (Table S7). As mentioned previously, there is no annotation for Pro^GGG^ and Asn^AUU^ in the bovine genome. Interestingly, the most frequent codon in the proline family pairs to the anticodon Pro^GGG^, which suggests that wobble base pairing can be used to compensate. Contrastingly, the most frequent codon in the asparagine family (Asn^AAC^) pairs to the only asparagine tRNA anticodon annotated in the bovine genome (Asn^GUU^). It is important to note that Asn^AAT^ was present in lower frequencies in the CDS of all treatments and tissues (Table S7).

**Fig. 5:**
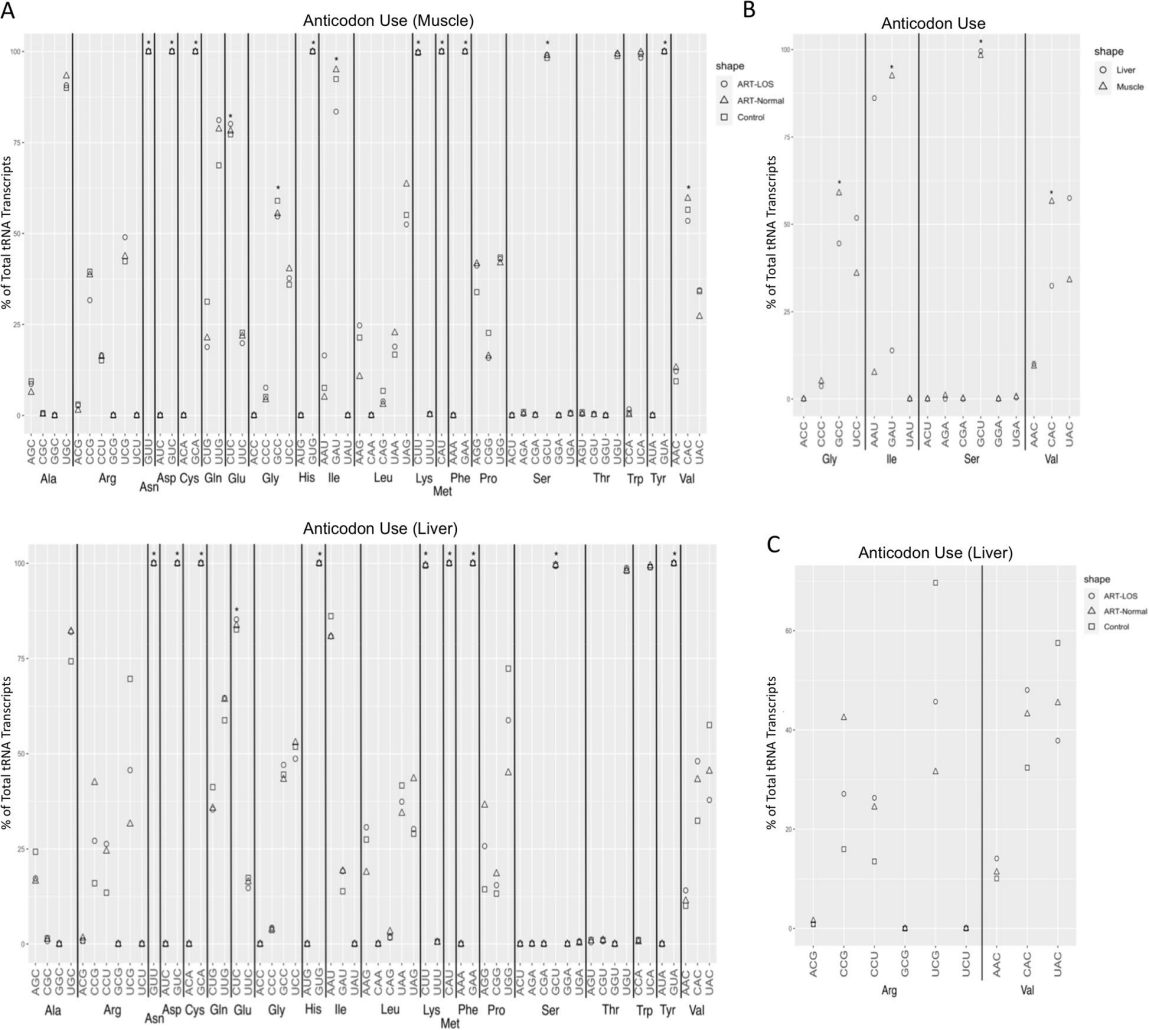
Anticodon use within an amino acid family. Each amino acid family is independent of one another. All isoacceptors within an amino acid family totals to 100% and the height of the respective dot indicates the most highly expressed tRNA for that family. Black * indicate correlated anticodon expression and codon usage in bovine genome. (**A**) Anticodon use across all amino acid families in ART-LOS, ART-Normal, and Control-AI in (top) muscle and (bottom) liver. (**B**) Anticodon use across select mature tRNA isoacceptors in the control individuals of muscle and liver tissues. (**C**) Anticodon use in a subset of mature tRNA isoacceptors across treatment groups

**Fig. 6:**
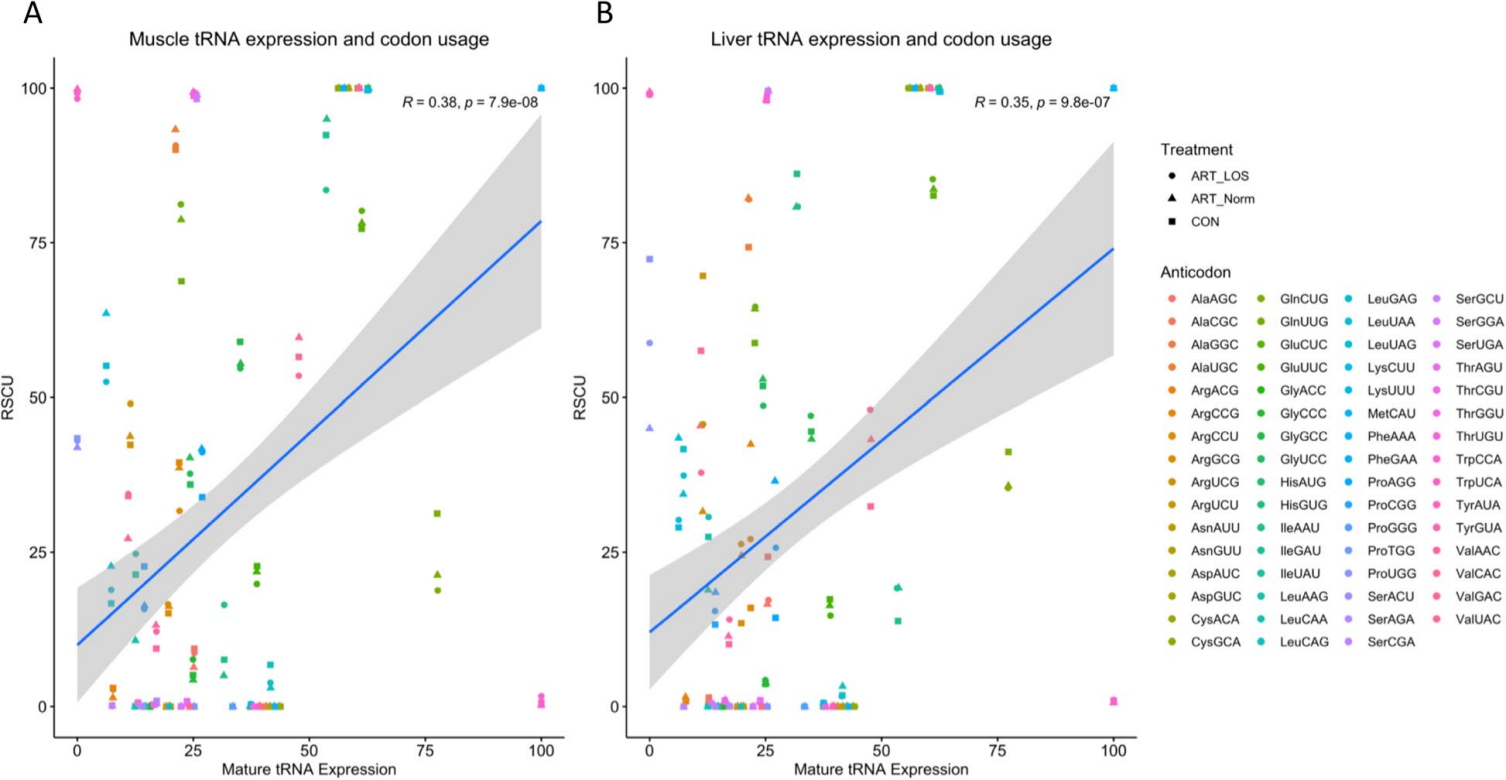
Correlation between relative synonymous codon usage (RSCU) and tRNA expression. All isoacceptors within each amino acid family total to 100% for RSCU and tRNA expression datasets in (**A**) muscle and (**B**) liver

From there, we sought to investigate the tRNA anticodon contribution within each amino acid family (Fig. **5**). Using dot plots, tRNA isoacceptors were grouped and the abundance within each amino acid family is represented as a percent of total tRNA transcripts. This allows us to rank the contribution of tRNAs charged with the same amino acid and identify preferences in anticodon availability. Furthermore, the tRNA expression data was compared to our codon usage analysis to identify a correlation between codon usage frequency and tRNA species concentration. Black asterisks indicate that the most highly expressed tRNA pairs to the most frequent codon in expressed transcripts (Fig. **5**A). Out of the 20 amino acid families, 13 and 10 had an anticodon that corresponded to the most frequent codon in muscle and liver. In Control-AI individuals, we investigated tissue specificity of codon: anticodon interactions (Fig. **5**B). For example, glycine, isoleucine, and valine all showed differences in the most highly expressed tRNA depending on tissue type. However, we did observe instances of the most highly expressed tRNA being shared between the two tissues (serine). Interestingly, the highest contributing tRNA isoacceptor in glycine, isoleucine, and valine correlated to the most frequent codon in one tissue, but not the other. This suggests that there is biased expression to potentially regulate protein synthesis and may reflect tissue-specific codon optimality. Furthermore, we examined treatment-specific anticodon use (Fig. **5**C). For example, the liver tissue showed changes in isodecoder contribution within Arginine and Valine families. In arginine, ART-Normal individuals had a higher abundance of Arg^CCG^ whereas Control-AI and ART-LOS individuals showed a higher contribution of Arg^UCG^. In valine, the Control-AI and ART-Normal groups display higher levels of Val^UAC^ compared to ART-LOS. Valine acts as an example of an isoacceptor family influenced by tissue type in Control-AI individuals (Fig. **5**B) and treatment type in phenotypically normal groups (Fig. **5**C). In the muscle, we observe small variations in tRNA expression but find no instances of a shift in the most highly expressed tRNA between treatment groups. In an effort to investigate if codon usage could be explained by tRNA availability, we performed a Pearson correlation analysis between RSCU values and tRNA expression (Fig. **6**). We found a modest positive correlation between codon usage and tRNA expression in bovine muscle (*R* = 0.38; *p*-value ≤ 0.05) and liver (*R* = 0.35; *p*-value ≤ 0.05). This statistically significant correlation is similar to values previously reported in mouse [39] and human[40], and suggests other elements contribute to codon usage in eukaryotes. This could indicate that important transcripts are enriched for codons that pair to highly expressed tRNAs. Therefore, fluctuations in tRNA abundance can demonstrate a mechanism, in which cells respond to tissue type or condition to regulate protein production through codon optimality.

## Discussion

Although ART induced manipulation of the cellular environment is known to alter the epigenome and gene regulation, the mechanisms in which this contributes to overgrowth is poorly understood and diagnosis is difficult because of variability in the presence of major clinical symptoms [41–44]. Whole genome bisulfite sequencing across tissues in cattle has shown tissue-specific methylation patterns [11]. Given that different developmental stages and aging in non-disease states can be associated with tRNA loci methylation, alterations in tRNA expression within tissues and epigenetic disorders could be explained by DMRs [12, 45]. Due to the dysregulation of transcripts and the presence of differently methylated regions in ART-LOS individuals, we suggest that the syndrome could be influenced at the translational level through fluctuations in tRNA availability [5, 46]. Here we evaluated the complexities of tRNA expression in the muscle and liver tissue of bovine using a method for efficient high throughput sequencing of full length mature tRNAs [47]. Through the employment of this sequencing method, we have improved adapter ligation efficiency and retained full length mature tRNAs during library preparation. We must acknowledge that post-transcriptional modifications are a source of stalling and read errors during reverse transcription (RT). This can result in truncated sequences and a reduced ability to quantify all tRNAs. Other methods have described demethylation treatments to remove RT-impairing modifications, inclusion of truncated cDNA bands, or used alkaline hydrolysis to break tRNAs into shorter fragments with fewer modifications [48, 49]. The application of these techniques could decrease bias and increase sensitivity for future tRNA studies.

Data analysis via PCA and RLE plots shows variation in tRNA expression across and within treatment groups in muscle and liver (Supplementary Fig. 1 & 2). These findings are in agreement with a previous study, in which tRNA isoacceptors did not tightly cluster in PCA plots of Archaea, Bacteria, and Eukarya [35]. Because of this diversity in tRNA expression, we found fewer statistically significant differentially expressed tRNAs (Table S5). However, the highest number of differentially expressed tRNAs were identified in the pairwise comparison between ART-Normal and ART-LOS in both tissues, which displayed the least variation in the PCA plots (Supplementary Fig. 1C, 2C). Through analysis of isodecoder gene copy numbers in several species, we addressed the evolutionary question of the redundancy of tRNA genes. We propose that a gene expansion event occurred, resulting in a series of duplications of tRNA genes and increased numbers in ruminant species (Fig. **1**A). This hypothesis is further supported through our observation of tRNA gene copy number conservation across cattle, goat, and sheep (Fig. **1**B). From there, we asked if the expression of certain tRNAs was influenced by tissue and treatment. Due to the redundancy of tRNA genes across the genome, we classified unique tRNAs by identifying sequences that were only expressed in the muscle or liver tissue within a particular treatment group, as well as those only expressed in Control-AI, ART-Normal, or ART-LOS within a particular tissue (Fig. **2**A-B; Table S4). While most tRNA sequences had shared expression across tissues and treatments, we successfully identified subsets of sequences that were specific to tissue and disease. These unique tRNA sequences could underlie tissue specific regulatory mechanisms. The availability of mature tRNAs in the cytoplasm as well as codon usage bias can directly modulate protein synthesis [24, 50–52]. In addition, tRNA derived fragments (tRFs) result from fragmentation of the mature tRNA. The presence of unique nucleotide sequences in a particular tissue or treatment may yield distinct tRF subtypes, resulting in targeting of mRNA transcripts by these sequence specific regulatory products. Furthermore, the ability of these unique tRNAs to specifically cleave may influence the regulation of genes important for maintenance of growth and development within a defined tissue or treatment [37, 53, 54]. Since numerous studies have reported variation in the expression of tRNA species in disease, we propose that specific tRNA loci are actively transcribed in at least one tissue and/or treatment but remain unexpressed in another [55–58]. Roughly half of human tRNA genes are transcriptionally silent or lowly expressed [16] and we identified that approximately 70% of bovine tRNA genes are unexpressed in muscle and liver. An increase in the percentage of unexpressed tRNAs could be contributed to the nearly three-fold increase in the number of annotated tRNA genes in bovine compared to human, or even that some of these genes may be tRNA pseudogenes [48]. While we did not find evidence of expression for a majority of tRNA genes in the genome, those expressed tRNAs represent isoacceptors for all amino acids and 50 of the expected 61 codons. This is concordant with the human cytoplasmic pool of tRNAs, in which only 48 isoacceptors code for the 20 amino acids [14]. An abundance of tRNA genes has also been suggested to play a role in genome structure [16]. For example, MT-tRNAs reside between mRNAs and rRNAs on polycistronic transcripts, which allows separation of mRNAs encoded in the mitochondria [59]. While cytoplasmic tRNAs differ from MT-tRNAs, both active and silent tRNA genes could punctuate DNA sequences to regulate gene expression and cellular function. Alternatively, perhaps some of these transcriptionally inactive tRNAs are expressed in other tissue types aside from those analyzed in this study or that these tRNAs remain unexpressed across homologous species [60, 61]. To confirm this hypothesis, tRNA expression in additional tissues and other ruminant species should be investigated.

After finding that a subset of tRNA genes were actively transcribed, we found a relatively weak relationship (*R* < 0.4, *p*-value < 0.05) between gene copy number and tRNA expression for both tissues (Fig. 3). This suggests that copy number does not dictate tRNA abundance and supports previous observations that tRNA isodecoder concentration is correlated to translationally optimal codons instead of copy number [62, 63]. As previously mentioned, we detected differentially expressed tRNAs and variations between treatment groups (Fig. **4**A-B,Table S6). Based on the pairing of tRNA anticodons to the codons within a transcript, highly or lowly abundant tRNAs could be used to predict translation speed of codons across tissues [39, 61, 64]. This balance between readily available tRNAs and codons in transcripts explains codon optimality in bovine, which acts as a means to monitor translational speed and affect mRNA stability [65, 66]. We also detected tissue-dependent isodecoders with no detected expression across all treatment groups in both tissues (Fig. **4**C-D). The absence of certain tRNA isodecoders can be rescued by the utilization of wobble base pairing, yet we could suggest this would result in alterations during protein synthesis. Our evaluation of anticodon contribution within an isoacceptor family showed conservation of highly abundant tRNA isodecoders in muscle and liver as well as differences in expression profiles across tissues and treatment types (Fig. **5**). Although the most frequent codon in the bovine and human genome was correlated in roughly half of the isoacceptor families, the degeneracy of the codons allows for translation to occur even if it is at an altered rate. We found clear preferences in tRNA expression for a given tissue and treatment group, demonstrating that tRNA levels are dynamic in both normal and overgrowth states (Fig. **5**B-5C). For example, isoacceptors for isoleucine display robust rearrangements, decreasing expression of one tRNA as another isoacceptor increases across Control-AI tissues. In addition, the switch in the highest contributing tRNA isodecoder between Control-AI and LOS individuals reveals the influence of the overgrowth phenotype on tRNA expression. Furthermore, we used RNA-seq data from muscle and liver tissue of day 105 Control-AI, ART-Normal, and ART-LOS bovine fetuses to perform a codon usage analysis (Table S7). We found that the RSCUs were the same regardless of treatments and tissues, which may be due to analyzing all expressed genes (≥ 1 TPM) in each group. Furthermore, a modest but significant correlation was observed between codon usage and tRNA expression. This could indicate that the frequency of codons in essential transcripts could be linked to tRNA availability and associated with varying translation rates. Further investigation is required to evaluate the impacts of tRNA expression on proteome composition. Overall, tRNA abundance could act as a source of genetic variation, which regulates protein production based on codon: anticodon interactions.

## Conclusion

Despite being thought to have pervasive expression, we have demonstrated the complexities of tRNA abundance. This study evaluated the active and silent states of tRNA genes within the bovine genome and also identified variation in the tRNA pool within different tissues and across naturally conceived, ART-normal, and ART-LOS individuals. Variation in the expression of tRNA genes could aid in reduced or increased translational efficiency of transcripts related to homeostatic maintenance in defined tissues, increased muscle mass and/or liver tumor cell proliferation. Furthermore, the presence of distinctive tRNAs in muscle and liver could support modulation of protein synthesis through the availability of tRNAs delivered to the ribosome or through targeting of mRNA transcripts by sequence specific tRFs. Our findings have detected that certain tRNA isodecoders within an amino acid family have the most predominant expression dependent on tissue and treatment. We can suggest there is a need to reconsider the consequences of synonymous mutations in the genome because of the relationship between tRNA availability, codon optimality, and translational stalling.

## Methods

### Animals and RNA isolation

We previously generated Day 105 Bos taurus indicus (B. t. indicus; Nelore breed) × Bos taurus taurus (B. t. taurus; Holstein breed) F1 fetal conceptuses [67]. Tissues were flash frozen in liquid nitrogen and stored at -80 °C until RNA extraction. Total RNA was extracted from skeletal muscle and liver tissues of F1 hybrid controls (artificial insemination; Control-AI), in vitro produced ART-Normal (similar weight as controls), and in vitro produced ART-LOS (body weight greater than 97th centile relative to controls) using TRIzol Reagent (Invitrogen, Carlsbad, CA) following the manufacturer’s instructions. Quality and concentration of the RNA samples was assessed using the Agilent TapeStation RNA ScreenTape (Agilent, Santa Clara, CA) and RNA integrity numbers (RIN) of all samples were > 7.4, suggesting high quality total RNA.

### Library preparation and sequencing

Mature tRNA library preparation from skeletal muscle and liver tissue was performed according to the YAMAT-seq protocol [47]. Total RNA samples were incubated at 37 °C for 40 min in 20 mM Tris–HCl (pH 9.0) to deacylate mature tRNAs, thus removing amino acids. Following the deacylation treatment, deacylated total RNA was purified with the RNA Clean & Concentrator-5 kit (Zymo Research, Irvine, CA). Concentration was assessed using the Agilent TapeStation RNA ScreenTape (Agilent, Santa Clara, CA). A 3’ adapter and DNA/RNA hybrid 5’ adapters were used to hybridize the different discriminator bases preceding the 3’ CCA tail on mature tRNAs: A (Y-5’-AD-A), G (Y-5’-AD-G), C (Y-5’-AD-C) and U (Y-5’-AD- U) [47]. 1 μg of deacylated RNA was mixed with 40 pmol of the 3’ adapter and 40 pmol of the four 5’ adapters (10 pmol each) and then incubated in a 9 μl reaction at 90 °C for 2 min. 1 μl of 10 × annealing buffer (50 mM Tris–HCl (pH 8.0), 5 mM EDTA, and 100 mM MgCl_2_) was added to the adapter/RNA mixture and annealed at 37 °C for 15 min. 10 μl of 1 × reaction buffer (10 μl 10 × buffer, 8.7 μl RNase free water and 0.3 μl T4 RNA ligase 2) was added to the mixture. The resulting mixture was incubated at 37 °C for 1 h and then 4 °C overnight. After annealing and ligation, the TruSeq® small RNA Library Preparation Kit (Illumina, Inc, San Diego, CA) was used for reverse transcription and library amplification via PCR. A unique indexed primer was used for each library sample. Following PCR, each library was run on a High Sensitivity DNA chip (Agilent, Santa Clara, CA) with expected peaks of approximately 200–240 bp. The amplified libraries were pooled in equal concentrations, run on a 6% Novex TBE PAGE gel with 1 × Novex TBE Buffer (Thermo Fisher Scientific, Waltham, MA) and stained with ethidium bromide. A size selection of 160 to 300 bp was performed on the gel via a UV transilluminator. Pooled libraries were gel purified and sequenced using Illumina NextSeq 500 System Mid-Output Kit (Illumina, Inc., San Diego, CA) by the OSU Genomics and Proteomics Center. The pooled libraries were sequenced on a single lane. There was an average of approximately 5 million and 4 million single-end reads of 150 bases acquired for each muscle and liver sample.

### Processing and mapping of tRNA Reads

The YAMAT adapter sequences (3’: GTATCCAGTTGGAATTCTCGGGTGCCAAGG; 5’: GTTCAGAGTTCTACAGTCCGACGATCACTGGATACTGGN) were removed from raw sequence reads with trimmomatic [68]. Reads at least 50 bp in length were retained. An index of the ARS-UCD1.2 genome was generated with hisat2-build and HISAT2 was used to align to the genome with the -dta-cufflinks option and -k 100 parameter [69]. Samtools was used to convert from SAM to BAM files and Samtools sort was used to sort each BAM file by gene locus. Featurecounts was used for read count estimation with the -s 1 parameter for strand specific data, -T 12 parameter to specify the number of threads, and -M to allow multi-mapped reads that align to the genome more than once. No mismatches were allowed. tRNA gene predictions in the Bos taurus genome were made by tRNAscan-SE to classify high confidence tRNA genes [33]. Read counting was performed at a feature level with parameter -t tRNA for read count estimation of nuclear and mitochondrial tRNA genes.

### Data analysis

EdgeR v 3.24.3 was used to conduct a differential expression analysis of the raw read counts of tRNAs [70]. Only tRNAs that had at least 5 counts per million in all of the control, or all of the ART-normal, or at least 2 ART-LOS were considered highly expressed and kept for DE analysis. Raw read counts were then normalized with RUVseq v1.16.1 [71]. Three separate differential expression tests were performed in both skeletal muscle and liver tissues: Control-AI vs ART-normal, ART-normal vs ART-LOS, and Control-AI vs ART-LOS. Differentially expressed tRNAs had a p-value and false discovery rate (FDR) of ≤ 0.05.

Principal component analyses (PCA) and relative log expression (RLE) plots were used with the plotPCA and plotRLE function of the DESeq2 package v1.22.2, respectively. Venny 2.1.0 (https://bioinfogp.cnb.csic.es/tools/venny/index.html) was used to produce all Venn diagrams. Samtools faidx and tRNA coordinates from the ARS-UCD1.2 genome annotation file were used to retrieve all tRNA sequences from the bovine assembly. tRNA data was merged at the level of the anticodon. Scatterplots depicting the relationship between tRNA expression and copy number were made with the ggplot function of the ggplot2 package. Ggplot2 was also used to produce stacked bar graphs depicting expressed and unexpressed isodecoders within the control group. The total number of gene copies was counted by the number of annotations in the GFF file for the reference genome. Log transformed CPM values were used to better distinguish differences in expression in bar graphs depicting tRNA expression levels in each treatment group. tRNAs that had no detectable expression in either tissue or in any treatment group were removed. The RSCU and tRNA expression values total to 100% across all of the isoacceptors in each amino acid family. Scatterplots (tRNA expression vs copy number; tRNA expression vs RSCU) were created with the ggscatter function of the ggpubr package to estimate the Pearson’s correlation coefficient and add colored regression lines corresponding to treatment group. The phylogenetic tree was generated using the software PhyloT v2 (https://phylot.biobyte.de) and based off of NCBI taxonomy. The interactive tree of life (iTOL v6) (https://itol.embl.de) was used for visualization of trees.

RNA-seq datasets for tissue-specific and treatment-specific codon usage analysis were retrieved from (NCBI Gene Expression Omnibus (GEO) accession numbers GSE63509). Additional RNA-seq data for the ART-Normal individuals is not yet available in the GEO database, but was provided by the Rivera Laboratory at the University of Missouri-Columbia. Genes were classified as expressed in a treatment group if they had ≥ 1 TPM in at least 2 replicates. We downloaded the coding sequences (CDSs) of each gene in the bovine genome (ARS-UCD1.2) from Ensembl Biomart version 104 [72]. Codon frequency, relative synonymous codon uses (RSCUs), and relative adaptiveness of a codon (RAC) values were calculated for each gene using the “Bio::Tools::CodonOptTable” module in the BioPerl package. Custom PERL scripts were used to average the values in each data set.

## Supplementary Information

**Additional file 1.**

**Additional file 2.**

**Additional file 3.**

**Additional file 4.**

## Data Availability

tRNA sequencing data reads are deposited in FASTQ format to the NCBI Sequence Read Archive database (SRA) under the BioProject accession number PRJNA764096.

## Abbreviations

ART: Assisted Reproductive Technologies
BWS: Beckwith-Wiedemann Syndrome
LOS: Large Offspring Syndrome
DMR: Differentially methylated regions
tRNA: Transfer RNA
tRF: TRNA derived fragment
SINE: Short interspersed repeated element
MT: Mitochondrial
AI: Artificial insemination
TPM: Transcript per million
RSCU: Relative synonymous codon use
RT: Reverse transcription
RIN: RNA integrity number
FDR: False discovery rate
PCA: Principal component analysis
RLE: Relative log expression
GEO: Gene expression omnibus
CDS: Coding sequence
RAC: Relative adaptiveness of a codon
SRA: Sequence read archive
