# Supplementary figures and images for: Characterization of tRNA expression profiles in large offspring syndrome
Source: BMC Genomics. 2022 Apr 7;23:273. doi: 10.1186/s12864-022-08496-7 (PMC8988405; doi:10.1186/s12864-022-08496-7)

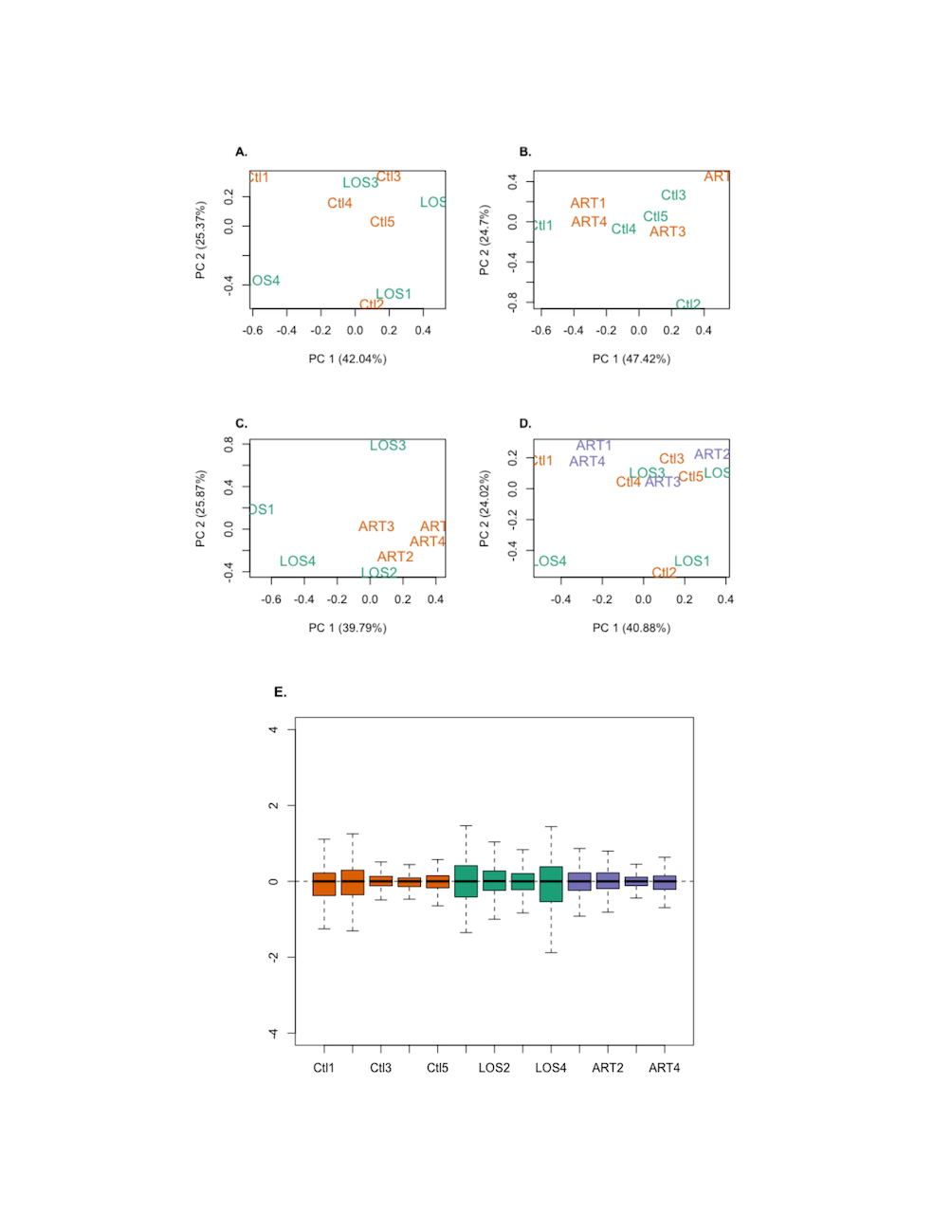

Supplement: Supplementary file 2 — Additional file 2. [file 12864_2022_8496_MOESM2_ESM.tiff]

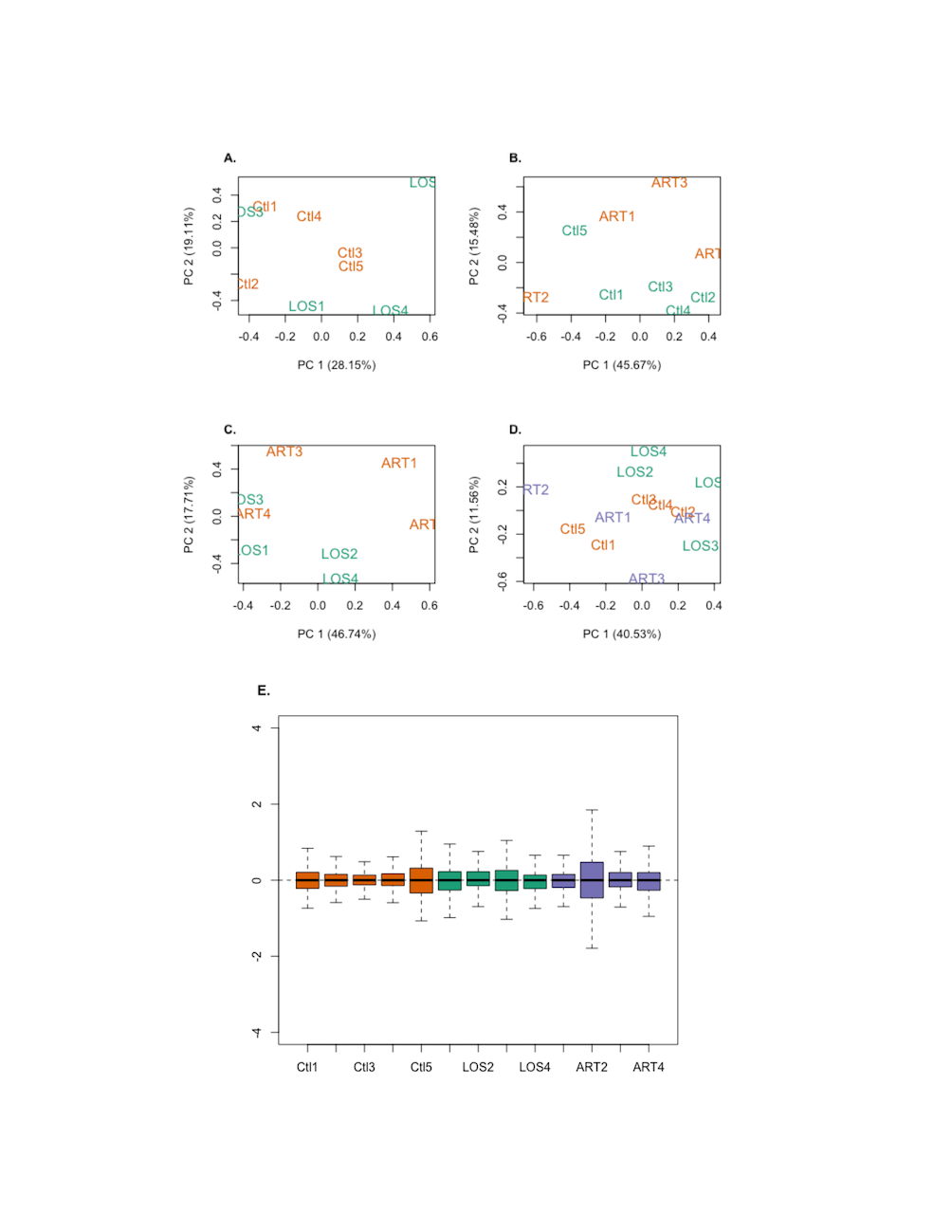

Supplement: Supplementary file 3 — Additional file 3. [file 12864_2022_8496_MOESM3_ESM.tiff]

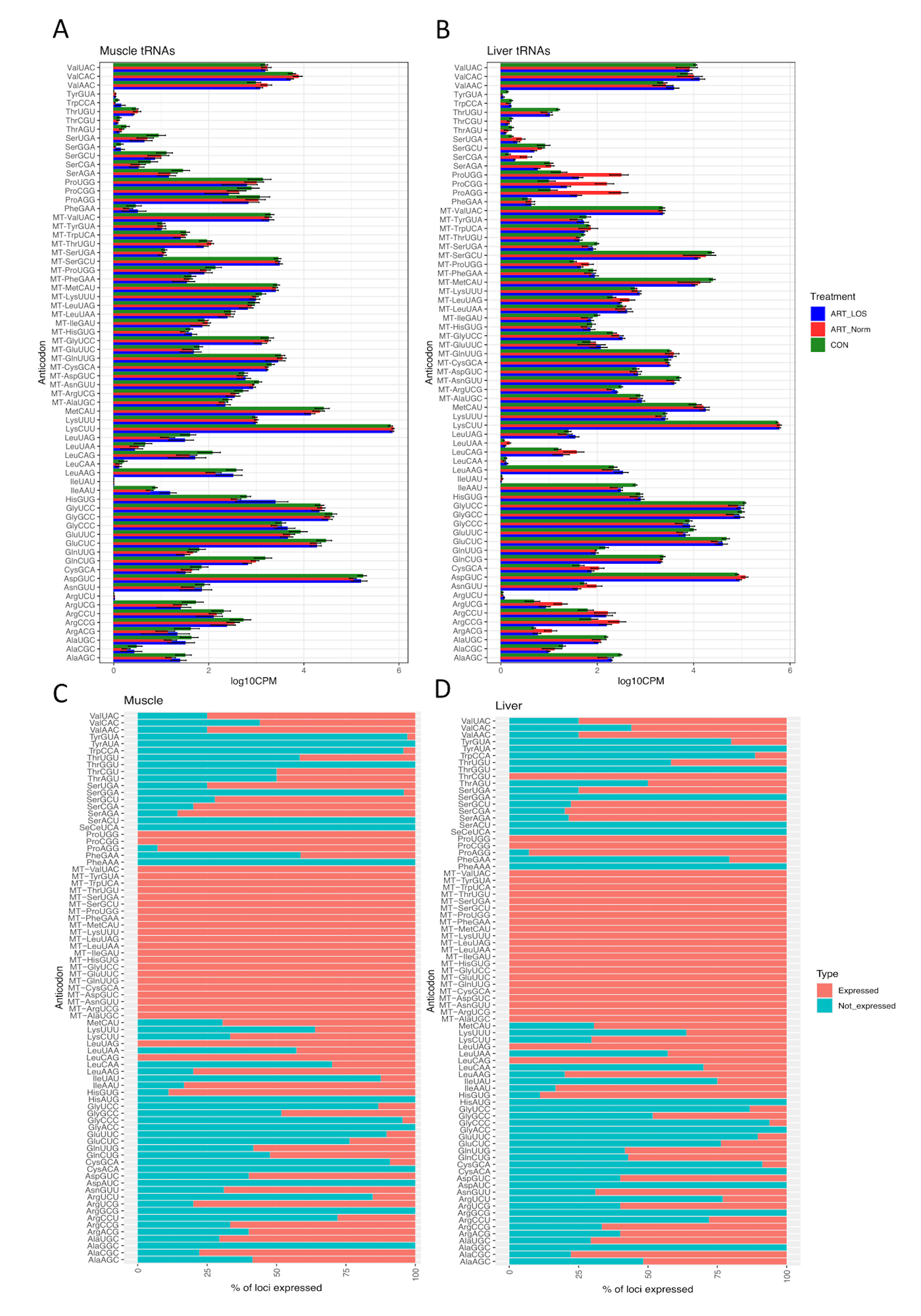

Supplement: Supplementary file 4 — Additional file 4. [file 12864_2022_8496_MOESM4_ESM.tiff]
